# Efficacy and Safety Profile of Combining Vandetanib with Chemotherapy in Patients with Advanced Non-Small Cell Lung Cancer: A Meta-Analysis

**DOI:** 10.1371/journal.pone.0067929

**Published:** 2013-07-04

**Authors:** Wei Tian, Wenping Ding, Sungkyoung Kim, Leizhen Zheng, Li Zhang, Xiaoping Li, Jianchun Gu, Lian Zhang, Minggui Pan, Siyu Chen

**Affiliations:** 1 Department of oncology, Xinhua Hospital Affiliated to Shanghai Jiao Tong University School of Medicine, Shanghai, P.R. China; 2 Department of Medical Oncology and Hematology, Kaiser Permanente Medical Center, Santa Clara, California, United States of America; Shanghai Jiao Tong University School of Medicine, China

## Abstract

**Objective:**

To evaluate the efficacy and safety profile of combining vandetanib with chemotherapy in patients with advanced non-small cell lung cancer (NSCLC).

**Methods:**

MEDLINE, EMBASE, Cochrane Central Register of Controlled Trials (CENTRAL), ASCO Abstracts, ESMO Abstracts, Wanfang Database, CNKI were searched. Eligible studies were the randomized clinical trials (RCTs) that compared the efficacy and safety profile of adding vandetanib to chemotherapy with single chemotherapy in patients with advanced NSCLC. The outcomes included overall survival (OS), progression-free survival (PFS), overall response rate (ORR) and toxicities. All meta-analysis were performed using Review Manager 5.1. The fixed-effect model weighted by the Mantel-Haenszel method was used. When considerable heterogeneity was found (*p*<0.1, or I^2^>50%), further analysis (subgroup analysis, sensitivity analysis or random-effect model) was performed to identify potential cause.

**Results:**

Results reported from 5 RCTs involving 2284 patients were included in the analysis. Compared to chemotherapy alone, the addition of vandetanib resulted in a significant longer PFS (HR 0.79 [0.72–0.87], *p*<0.00001) and a higher ORR (RR 1.75 [1.43–2.15], *p*<0.00001), but failed to show advantage on OS (HR 0.96 [0.87–1.06], *p* = 0.44).

**Conclusion:**

Vandetanib has activity in NSCLC. Identification of predictive biomarkers is warranted in future trials to select a subset of patients with advanced NSCLC who may benefit from vandetanib.

## Introduction

Lung cancer is the leading cause of cancer death world wide, with approximately 221,130 new cases in the United States in 2011, and 85% were non-small cell lung cancer (NSCLC) [1]. Although platinum-based doublet chemotherapy is the current standard treatment for patients with advanced NSCLC, median survival time has been no more than 10 months [2].

Agents targeting the vascular endothelial growth factor (VEGF) and epidermal growth factor receptor (EGFR) signaling pathways have been clinically validated in patients with advanced NSCLC [3], [4]. Moreover, EGFR is known to regulate the expression of VEGF, and increased VEGF expression is associated with resistance to EGFR inhibition [5], [6]. This suggests that a rational therapeutic approach would be combining inhibition of both EGFR and VEGFR signaling by using one single multi-targeted agent without increasing toxicity.

Over the past several years, a number of RCTs have been conducted to investigate the efficacy of adding vandetanib, a once-daily oral anticancer agent that targets VEGFR, EGFR and RET (rearranged during transfection) signaling [7], [8], to standard chemotherapy in patients with advanced NSCLC, but with diverse results. It is not clear if this type of combining a targeted therapeutic with chemotherapy provides clinical benefit. Therefore, we have undertaken this meta-analysis to evaluate the available evidence from the relevant RCTs. We will discuss the combined effects, their potential clinical applications and the future directions in this field.

## Methods

### Search Strategy

We have collected the eligible trials by searching the MEDLINE, EMBASE, Cochrane Central Register of Controlled Trials (CENTRAL), ASCO Abstracts, ESMO Abstracts, Wanfang Database, and CNKI up to October 2012. The Cochrane Highly Sensitive Search Strategy for identifying randomized controlled trials in MEDLINE (Ovid format) was used, as shown in **Table 1**. And the MEDLINE search strategy was adapted in other databases.

**Table 1:** Search strategy for MEDLINE (Ovid format) used in this Meta-analysis.

Search steps used for this Meta-analysis	
1.randomized controlled trial.pt.	16.(lung adj5 tumor$).mp.
2.controlled clinical trial.pt.	17.(lung adj5 tumour$).mp.
3.randomized.ab.	18.or/12–17
4.placebo.ab.	19.vandetanib.tw.
5.drug therapy.fs.	20.unresect$.tw.
6.randomly.ab.	21.inopera$.tw.
7.trial.ab.	22.advanc$.tw.
8.groups.ab.	23.unopera$.tw.
9.1 or 2 or 3 or 4 or 5 or 6 or 7 or 8	24.(non adj5 resect$).tw.
10.human.sh.	25.nonresect$.tw.
11.9 and 10	26.or/20–25
12.exp lung neoplasms/	27.19 and 26
13.(lung adj5 cancer$).mp.	28.18 and 27
14.(lung adj5 neoplasm$).mp.	29.28 and 11
15.(lung adj5 carcinoma$).mp.	

All the randomized controlled trials on vandetanib for advanced NSCLC were collected and identified. All reference lists from trials selected by electronic searching to identify further relevant trials were scanned. We have also searched published abstracts from conference proceedings of the American Society for Clinical Oncology (ASCO) and the European Society for Medical Oncology (ESMO).

### Inclusion Criteria

Eligibility criteria: (1) Type of participants: adults with previously treated or untreated locally advanced (stage IIIB) or metastatic (stage IV) NSCLC. (2) Type of study: studies had to be RCTs comparing the efficacy and safety profile of adding vandetanib to chemotherapy with single chemotherapy in patients with advanced NSCLC. This included the usage of any dosage and schedules of vandetanib as first or second line therapy. (4) Type of publication: All full papers on original data were included. Abstracts or unpublished data were also included if sufficient information on study design, characteristics of participants, interventions and outcomes was available and if full information and final results were confirmed by the first author.

### Exclusion Criteria

We excluded quasi-randomized studies that were considered to possess insufficient quality. Cross-over studies were excluded in order to assess the overall treatment effect on survival.

### Data Extraction and Quality Assessment

Two reviewers independently extracted the data from all included studies. Types of outcome measure included OS, PFS, ORR and toxicities. Only the most frequent events of toxicities were analyzed. We used the methods of summarizing hazard ratios (HRs) of time-to-event data (OS and PFS) provided by Jayne F Tierney *et al*. [9]. The HRs of time-to-event data (OS and PFS) were extracted from the original studies or accounted from the reported number of events and the corresponding *p*-value of the log-rank statistics, or by reading off survival curves. We assessed methodological quality of the studies using the Jadad score [10]. We graded each parameter of trial quality as full score (5), high score (≥3), and low score (≤2). We used the name of the first author and the year of publication of the article for identification.

### Statistical Analysis

All meta-analysis were performed using Review Manager 5.1. Time-to-event outcomes were compared using a hazard ratio (HR). Dichotomous data were compared using a risk ratio (RR). 95% confidence intervals (CI) were calculated for each estimate and presented in forest plots. Statistical heterogeneity in the results of the trials was assessed by the chi-square test, and expressed by the I^2^ index, as provided by Higgins *et al*. [11]. The fixed-effect model weighted by the Mantel-Haenszel method was used. When considerable heterogeneity was found (*p*<0.1, or I^2^>50%), further analysis (subgroup analysis, sensitivity analysis or random-effect model) was performed to identify the potential cause.

## Results

### Study Identification

**Figure 1:**
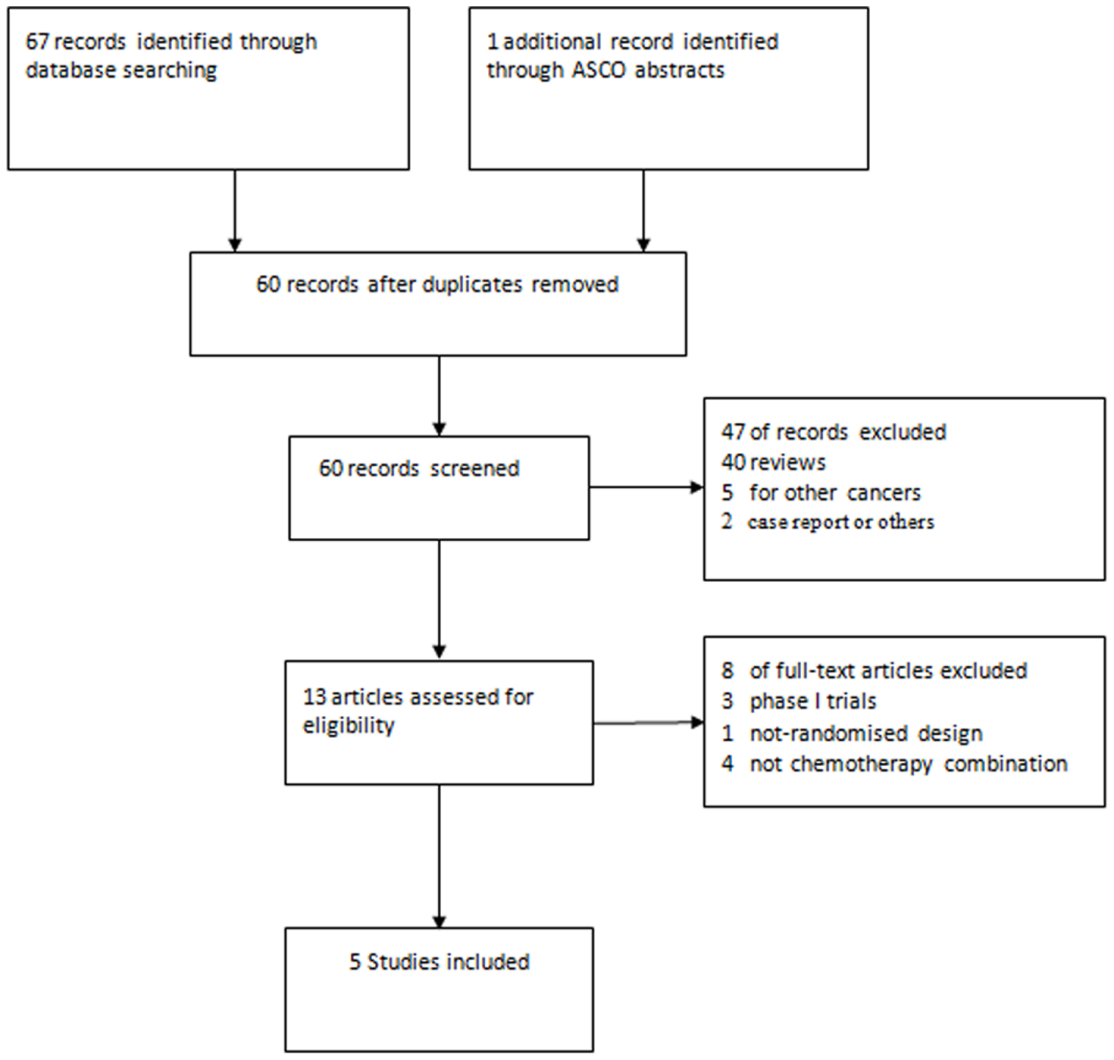
Flow chart for identification and inclusion of trials for this meta-analysis.

Our systematic search screened 67 trials, and found 5 publications related to 5 randomized clinical trials (2,284 patients) that compared chemotherapy with or without vandetanib in patients with advanced NSCLC [12]–[16]. These 5 publications included 4 full papers [12]–[15] and 1 conference abstract from ASCO annual meeting [16]. Other potential eligible studies were single-armed or no chemotherapy combination and were therefore excluded. Three phase II [12], [13], [16] and two phase III [14], [15] trials were included. There was consistency by the reviewers on the identification of studies and the data extraction. The PRISMA Checklist and Flow Diagram for the studies was shown in **PRISMA Checklist S1**, **PRISMA Flow Diagram S1** and **Figure 1**.

### Characteristics of Included Studies

Characteristics of the included trials were provided in **Table 2**. And methodological details potentially related to bias of the 5 trials were provided in **Table 3**. All the 5 trials included illustrations regarding randomization, and 2 of the trials described the detailed methods used for randomization [14], [15]. Thus, all trials were described as 1 or 2 score based on randomization criteria. All trials described the use of double-blind methodology. Three trials reported detail information of withdrawals [12], [14], [15]. All the 5 trials were graded as high score (≥3), and were finally included in the analysis.

**Table 2:** Characteristics of the five eligible randomized trials in this meta-analysis.

Author and year	Phase	Therapy line	Regimens (per arm)	Stage	Patients enrolled	Male (%)	Median age	Smoking (%)	Locally advanced(%)	WHO PS 0(%)	Squamous (%)	Ethnic white (%)	Primary endpoint
Heymach *et al* 2007	II	Second	Van(100 mg)+Doc	IIIB/IV	42	50	61	83	31	33	29	NR	PFS
			Van(300 mg)+Doc		44	57	60	91	20	36	32	NR	
			Placebo+Doc		41	66	58	90	32	37	27	NR	
Heymach *et al* 2008	II	First	Van(300 mg)	IIIB/IV	73	67	63	75	14	30	22	NR	PFS
			Van(300 mg)+Pac+Car		56	70	60	77	12	45	20	NR	
			Placebo+Pac+Car		52	71	59	79	10	31	29	NR	
Herbst *et al* 2010	III	Second	Van(100 mg)+Doc	IIIB/IV	694	72	59	77	14	36	27	59	PFS
			Placebo+Doc		697	68	59	75	15	34	23	60	
De Boer *et al* 2011	III	Second	Van(100 mg)+Pem	IIIB/IV	256	62	60	78	14	41	21	77	PFS
			Placebo+Pem		278	62	60	81	17	41	22	78	
Cesare *et al* 2012	II	First	Van(100 mg)+Gem	IIIB/IV	61	NR	NR	NR	NR	NR	NR	NR	PFS
			Placebo+Gem		63	NR	NR	NR	NR	NR	NR	NR	

PS: Performance Status. Van: Vandetanib. Doc: Docetaxel. Pac: Paclitaxel. Car: Carboplatin. Pem: Pemetrexed. Gem: Gemcitabine. NR: NO Report.

**Table 3:** Methodological details potentially related to bias of the 5 trials.

Author and year	Random	Blind	Allocation concealment	Withdraw description	ITT analysis	Multicenter	Jadad score
Heymach *et al* 2007	Yes	Yes	NC	NC	Yes	Yes	3
Heymach *et al* 2008	Yes	Yes	NC	Yes	Yes	NC	4
Herbst *et al* 2010	Yes	Yes	Yes	Yes	Yes	Yes	5
De Boer *et al* 2011	Yes	Yes	Yes	Yes	Yes	Yes	5
Cesare *et al* 2012	Yes	Yes	NC	NC	NC	NC	3

ITT: Intend-to-treat. NC: No Clear.

### Overall Survival

**Figure 2:**
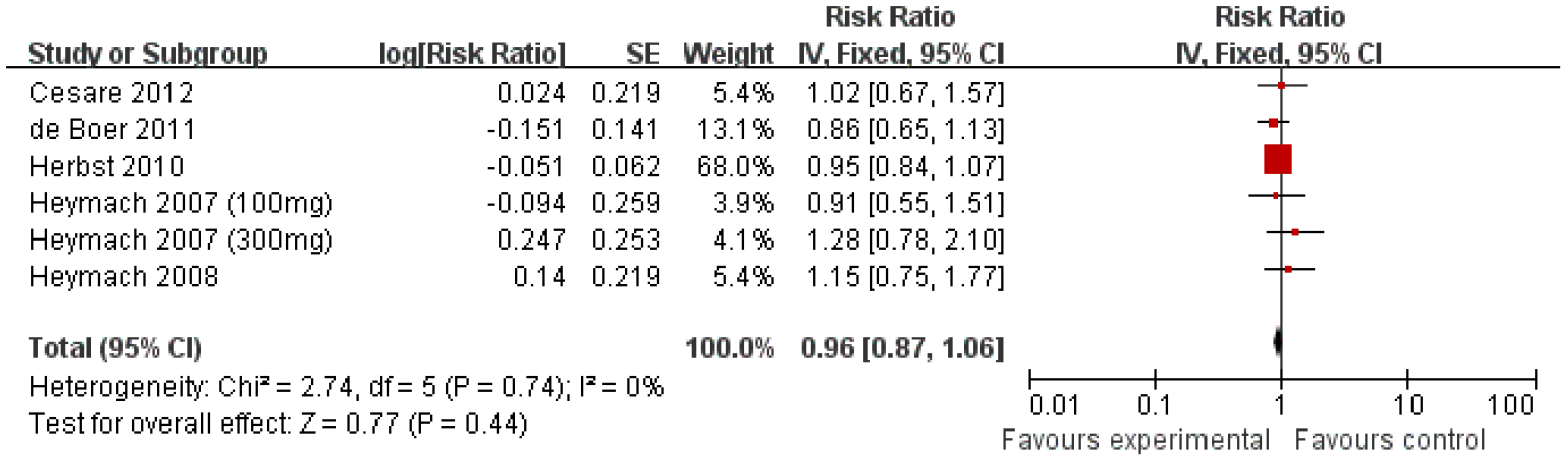
Comparison of OS between addition of vandetanib to chemotherapy and chemotherapy alone.

**Figure 3:**
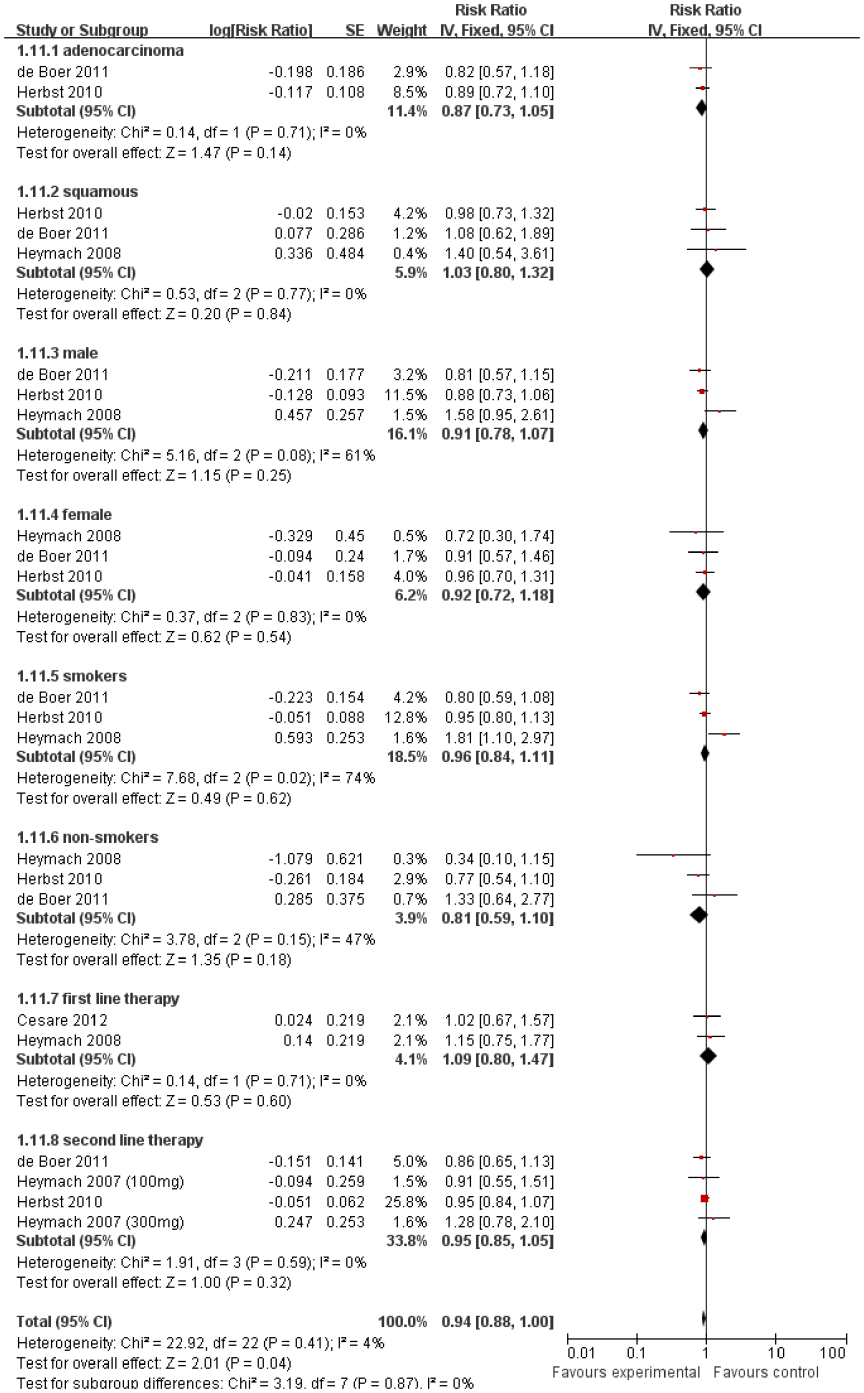
Subgroup analyses about the comparison of OS between addition of vandetanib to chemotherapy and chemotherapy alone.

The impact of vandetanib treatment on OS was extracted directly from published data of the 5 included trials. None of the 5 trials reported statistically significant improvement on OS. Meta-analysis showed that, the combination of vandetanib and chemotherapy resulted in no statistically improvement on OS compared with chemotherapy alone (HR 0.96 [0.87–1.06], *p* = 0.44), without apparent heterogeneity among the studies (*p* = 0.74, I^2^ = 0%) (**Figure 2**). Based on its lack of efficacy on OS in unselected patients, we took further subgroup analyses to define potential groups that may potentially benefit from vandetanib. Exploratory subgroup analysis defined by histology (adenocarcinoma or squamous), sex (male or female), smoking status (smokers or nonsmokers) and therapy line (first or second line therapy), showed similar results, without statistical significance in all the subgroups (**Figure 3**). When apparent heterogeneity was found in the subgroup of male (I^2^ = 61%) and smokers (I^2^ = 74%), random-effect model was used. However, this did not change the final results of the analyses that showed no statistical significances.

### Progression Free Survival

**Figure 4:**
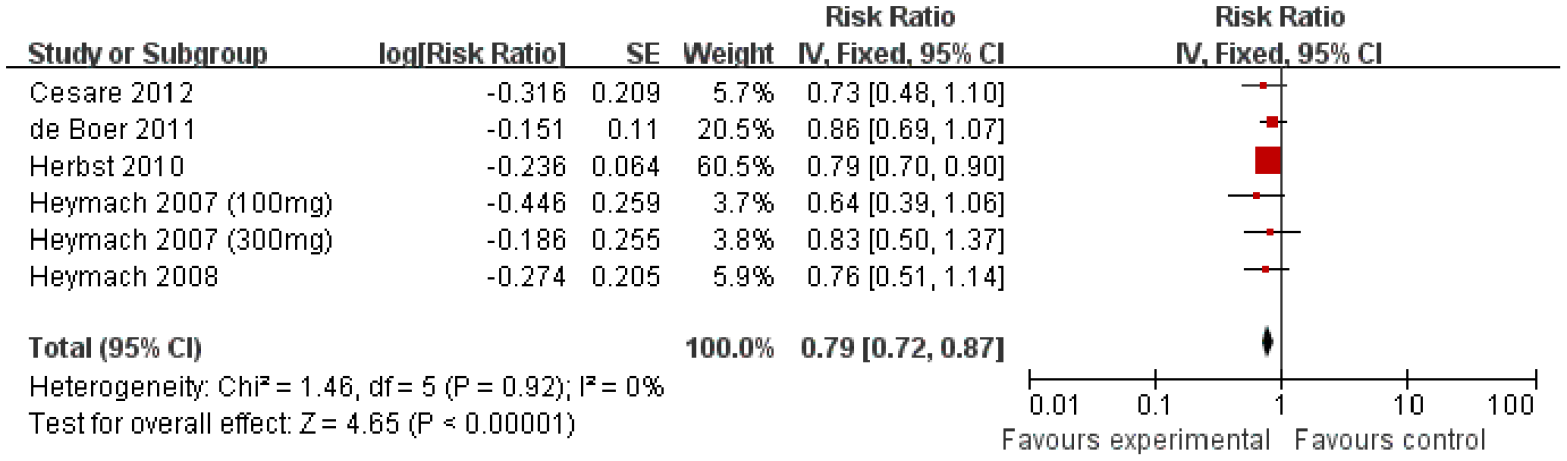
Comparison of PFS between addition of vandetanib to chemotherapy and chemotherapy alone.

All the 5 trials reported outcome of PFS as the primary endpoint. Compared to chemotherapy alone, the combination of vandetanib and chemotherapy resulted in statistically significant improvement on PFS (HR 0.79 [0.72–0.87], *p*<0.00001), without apparent heterogeneity among the studies (*p* = 0.92, I^2^ = 0%) (**Figure 4**).

### Overall Response Rate

**Figure 5:**
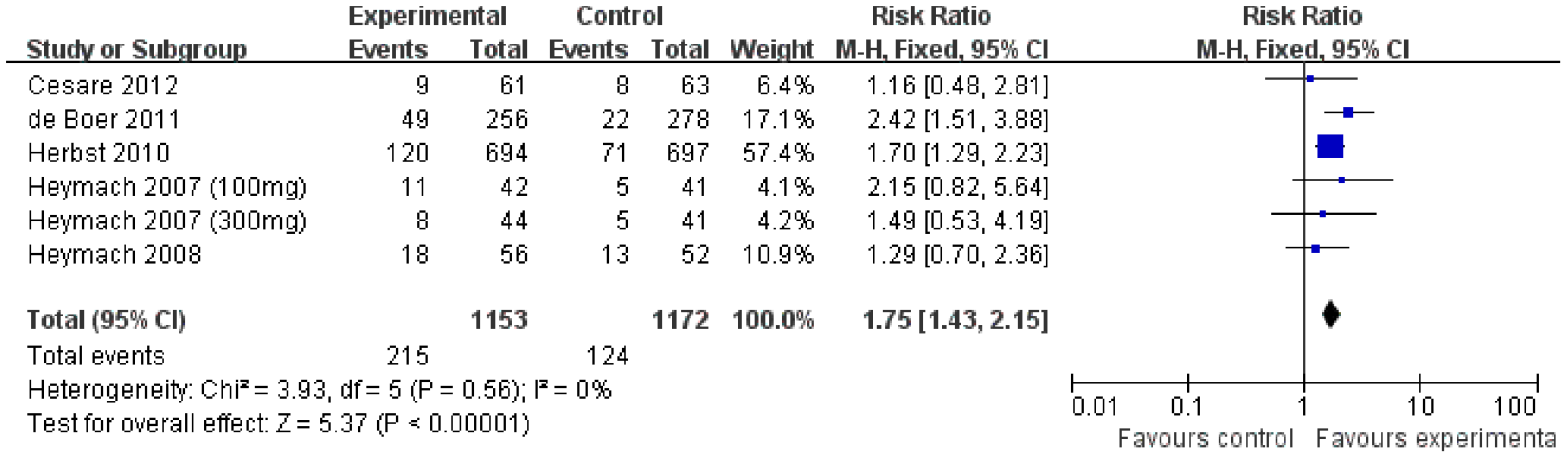
Comparison of ORR between addition of vandetanib to chemotherapy and chemotherapy alone.

All the 5 trials included in the analysis reported ORR. The combination of vandetanib and chemotherapy significantly improved the ORR (RR 1.75[1.43–2.15], *p*<0.00001), without apparent heterogeneity among the studies (*p* = 0.56, I^2^ = 0%) (**Figure 5**).

### Toxicities

**Figure 6:**
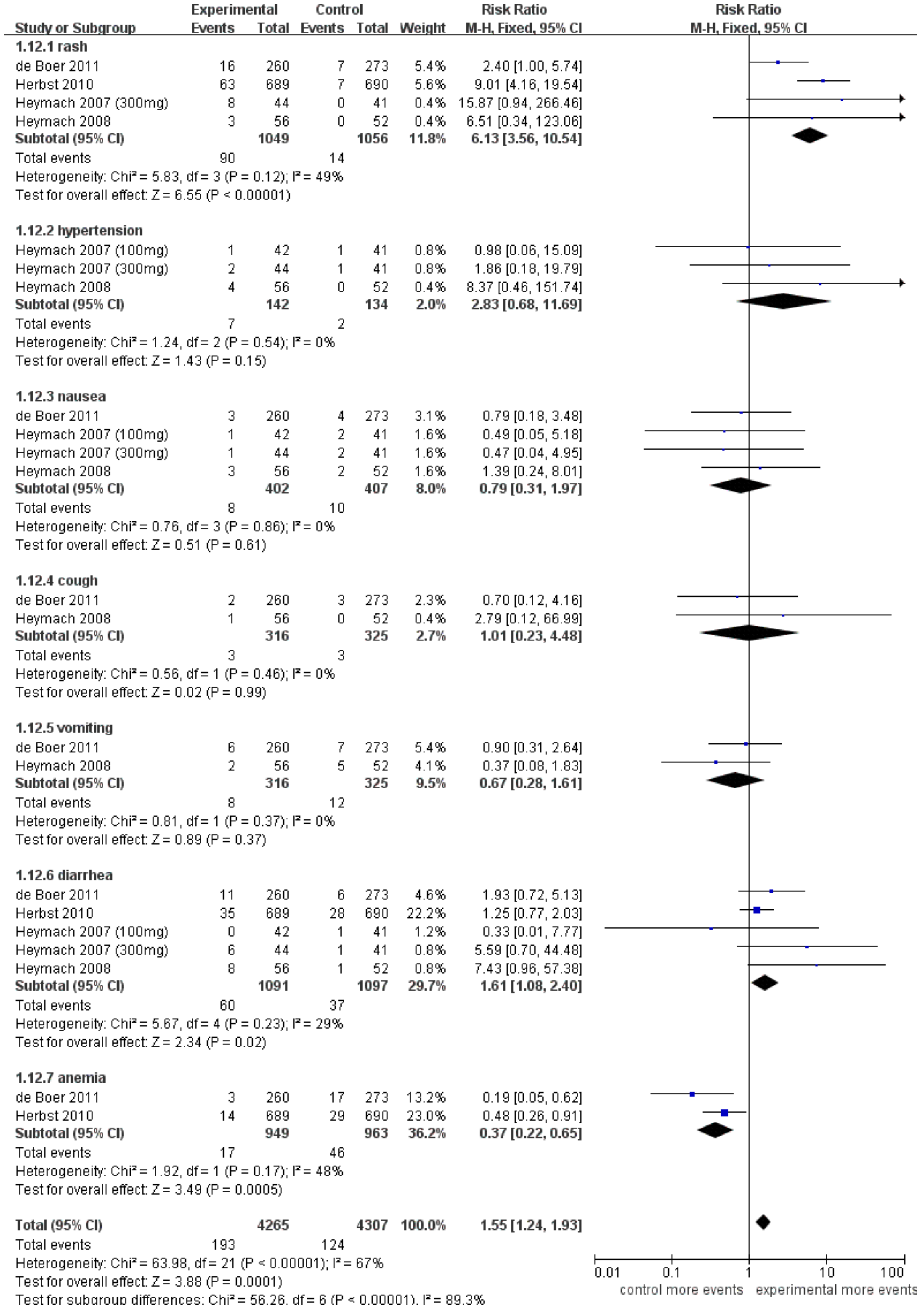
Comparison of grade ≥3 toxicities between addition of vandetanib to chemotherapy and chemotherapy alone.

**Figure 7:**
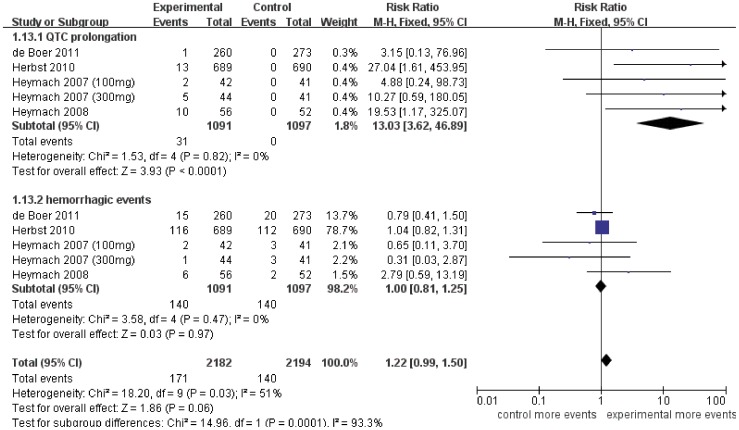
Comparison of QTc prolongation and hemorrhagic events of all grades between addition of vandetanib to chemotherapy and chemotherapy alone.

The outcome of the toxiticies with grade ≥3 for addition of vandetanib to chemotherapy was assessed. Only certain toxicities were consistently described in the 5 articles. We assessed the toxicities of rash and cough mainly caused by targeting EGFR, toxicity of hypertension mainly caused by targeting VEGFR, and other common toxicities occurred in the routine chemotherapy procedure, for example, the diarrhea, nausea, vomiting and anemia. The analysis showed that the grade ≥3 toxicities increased with the addition of vandetanib were rash (RR 6.13 [3.56–10.54], *p*<0.00001) (*p* = 0.12, I^2^ = 49%) and diarrhea (RR 1.61 [1.08–2.40], *p* = 0.02) (*p* = 0.23, I^2^ = 29%). The other toxicities including hypertension (RR 2.83 [0.68–11.69], *p* = 0.15) (*p* = 0.54, I^2^ = 0%), cough (RR 1.01 [0.23–4.48], *p* = 0.99) (*p* = 0.46, I^2^ = 0%), nausea (RR 0.79 [0.31–1.97], *p* = 0.61) (*p* = 0.86, I^2^ = 0%) and vomiting (RR 0.67 [0.28–1.61], *p* = 0.37) (*p* = 0.37, I^2^ = 0%) showed no statistically significant difference. Interestingly, the addition of vandetanib showed a significantly reduced incidence of anemia (RR 0.37 [0.22–0.65], *p* = 0.0005) (*p* = 0.17, I^2^ = 48%) (**Figure 6**). As QTc prolongation and hemorrhagic events of all grades were also important side effects of TKI targeting VEGFR, we took another analysis of these events as well. The analysis showed that QTc prolongation of all grades increased with the addition of vandetanib (RR 13.03 [3.62–46.89], *p*<0.0001) (*p* = 0.82, I^2^ = 0%). And hemorrhagic events of all grades showed no statistical difference (RR 1.00 [0.81–1.25], *p* = 0.97) (*p* = 0.47, I^2^ = 0%) (**Figure 7**).

### Publication Bias

**Figure 8:**
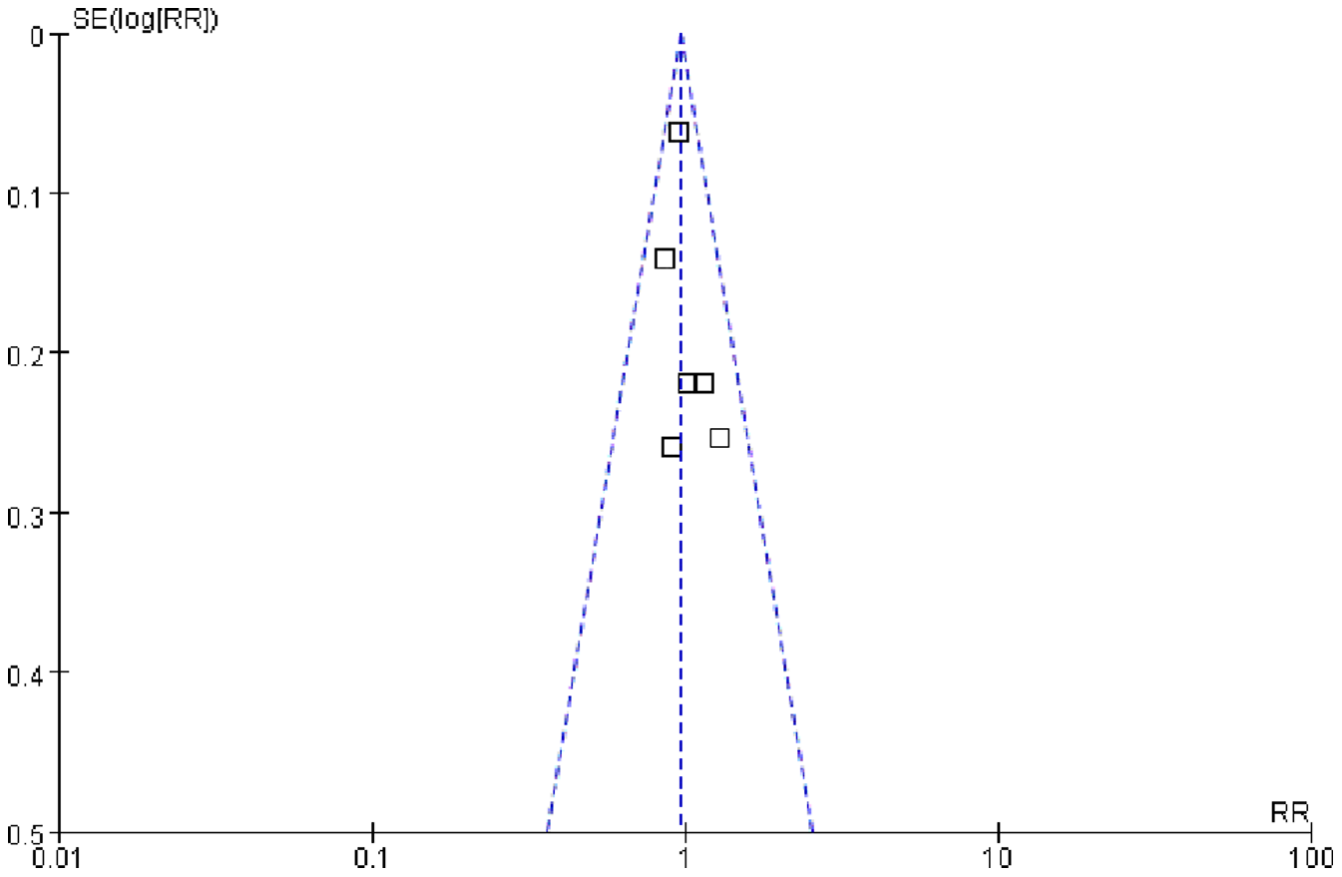
Funnel plot to assess for evidence of publication bias.

To minimize the potential of publication bias, we used the highly sensitive search strategy to identify the relevant trials. Furthermore, the papers were collected strictly according to the inclusion criteria and publication bias was detected by funnel plot. No apparent publication bias was found in the analysis (**Figure 8**).

## Discussion

Anticancer therapeutics that specifically target the well-defined signaling pathways important for cancer cell proliferation, invasion and metastasis such as EGFR or VEGFR pathway have shown promising clinical benefit in the treatment of advanced NSCLC [3], [4]. Furthermore, EGFR is known to regulate the expression of VEGF, and the resistance to tyrosine kinase inhibitors (TKIs) targeting EGFR may be partly associated with a rise in both host and tumor-derived VEGF [5], [6]. These were the basis for vandetanib, a once-daily oral anticancer agent that targets VEGFR, EGFR and RET signaling, to be combined with chemotherapy for advanced NSCLC in the clinical trials.

Our meta-analysis showed that addition of vandetanib to chemotherapy increased ORR and PFS, but did not improve OS in patients with advanced NSCLC. The results were consistent with the recently published meta-analysis on this subject conducted by Xiao YY *et al*. [17]. However, the clinical trials included in our analysis are more complete. Because the case volume in the Herbst *et al*. trial was the largest (occupied approximately 61% among the five RCTs), it led to 68%, 61%, and 57% relative weight in the OS, PFS, and ORR analysis respectively. However, when we performed additional analysis with the subtraction of the Herbst *et al*. trial data, the overall results remained similar [HR for OS was 0.99 [0.83–1.18], *p* = 0.88 (*p* = 0.62, I^2^ = 0%), HR for PFS was 0.80 [0.68–0.93], *p* = 0.005 (*p* = 0.84, I^2^ = 0%), the RR for ORR was 1.82 (1.34–2.48), *p* = 0.0001, (*p* = 0.42, I^2^ = 0%)]. Therefore, the weight of the Herbst *et al*. trial did not impact the overall results.

The most frequently reported adverse effect from vandetanib treatment was rash. Side effects caused by vandetanib, and particularly rash, appeared to be more frequent at higher doses. The meta-analysis conducted by Rosen *et al*. showed that patients who received vandetanib 300 mg had a significantly increased risk of developing all-grade rash in comparison with controls, with a relative risk of 2.43 (95% CI, 1.37–4.29; *p* = 0.002) [18]. Our meta-analysis showed that 100mg vandetanib could also increase the risk of grade≥3 rash (RR 5.77 [3.32–10.04], *p*<0.00001). The risk of grade≥3 diarrhea was also increased with the treatment of all dosage vandetanib. But the treatment of 100 mg vandetanib showed no statistically difference (RR 1.50 [0.99–2.26], *p* = 0.05). The prolongation of QTc by vandetanib requires further post-marketing surveillance.

Interestingly, we found that anemia was mitigated in the combination arm. But definitive conclusions could not be drawn because only 2 trials included in this analysis reported the side effect of anemia [14], [15]. The explanation of the reducted incidence of anemia treated with vandetanib might be that inhibition of VEGF signaling enhanced erythropoiesis through hypoxia induced factor (HIFa), which had been confirmed in preclinical models [19]. Awareness of these adverse events is critical for clinicians to ensure the best possible clinical benefit.

OS is the gold standard endpoint for clinical improvement in cancer patients. Our meta-analysis showed no improvement on OS, but significant on PFS. The rapid emergence of resistance to vandetanib may be responsible for this discrepancy between OS and PFS, and the ability to overcome drug resistance can obviously change patient outcome outcome and is an important future endeavor. The significant improvement on PFS suggests that vandetanib has activity in NSCLC, and there may be a subgroup of patients who could benefit from this drug which is currently approved by FDA for treating advanced medullary thyroid cancer. Subgroup analyses as defined by histology (adenocarcinoma or squamous), sex (male or female), smoke status (smokers or nonsmokers) and therapy line (first or second line therapy) did not show significant difference in OS. This indicates that there is a critical need for the identification of biomarkers for patients likely to benefit from vandetanib.

Hanrahan *et al*. found that, patients with low baseline plasma VEGF treated with vandetanib 100 mg/d and docetaxel appeared to have longer PFS and OS compared with those treated with docetaxel alone, whereas patients with high baseline VEGF showed similar treatment outcomes in both arms, but no definitive conclusions on the role of VEGF as a predictive biomarker for benefit from vandetanib could be drawn from this study because of its limitation [20]. However, the prognostic value of baseline plasma VEGF should be evaluated in the future clinical trials.

Furthermore, EGFR and KRAS are the most frequently mutated proto-oncogenes in NSCLC [21]. TKIs targeting EGFR have become important therapeutic options for patients with advanced NSCLC, patients whose tumors harboring a classic EGFR mutation or ALK (anaplastic lymphoma kinase) translocation can substantially benefit from erlotinib or gefitinib [22]–[24]. Whethere or not EGFR and ALK mutations can predict the benefit of vandetanib need to be investigated. Using KRAS mutation status for selecting treatment with EGFR-TKIs remains controversial. A meta-analysis of 22 studies conducted by Mao *et al*. identified KRAS mutation as a negative predictive biomarker for EGFR-TKI treatment in patients with NSCLC [25]. However, Guan *et al*. found that though KRAS mutation was a factor for poor prognosis, but not an independent predictor of response to EGFR-TKIs or chemotherapy in patients with lung cancer [26]. The relationship of KRAS mutation status and from the benefit of vandetanib treatment remains to be clarified.

Several limitations exist in this meta-analysis. First, although the publication bias was not found by funnel plots, the small number of the trials limited the power of the analysis. Second, one study we identified was reported in an abstract form only [16], which made it difficult to extract complete data for analysis, though this study was unlikely to change the overall results because of its small sample size. Furthermore, all the trials included in this analysis used PFS as primary end point. The only trial, conducted by de Boer *et al*., had a separate survival follow-up analysis [15].

In conclusion, Vandetanib has shown activity in NSCLC. The identification of predictive biomarkers is warranted in future trials to select a subset of patients with advanced NSCLC who may benefit from vandetanib.

## Supporting Information

Prisma Checklist S1 (DOC)

PRISMA Flow Diagram S1 (DOC)
