# Supplementary material for: Efficacy and Safety Profile of Combining Vandetanib with Chemotherapy in Patients with Advanced Non-Small Cell Lung Cancer: A Meta-Analysis
Source: PLoS One. 2013 Jul 4;8(7):e67929. doi: 10.1371/journal.pone.0067929 (PMC3701636; doi:10.1371/journal.pone.0067929)
Supplement: PRISMA Flow Diagram S1 — (DOC) [file pone.0067929.s002.doc]

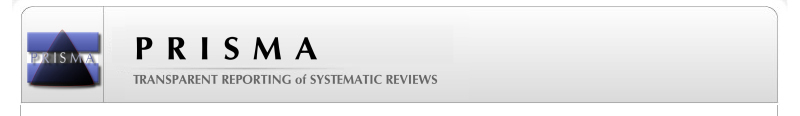
**PRISMA 2009 Flow Diagram**

**Screening**

**Included**

**Eligibility**

**Identification**

67 records identified through

database searching

1 additional record identified

through ASCO abstracts

60 records after duplicates removed

60 records screened

13 articles assessed for eligibility

8 of full‐text articles excluded

3 phase I trials

1 not-randomised design

4 not chemotherapy combination

5 Studies included

47 of records excluded

40 reviews

5 for other cancers

2 case report or others
